# Overall and diagnosis-specific sickness absence and disability pension in colorectal cancer survivors and references in Sweden

**DOI:** 10.1007/s11764-021-01017-7

**Published:** 2021-03-16

**Authors:** Luisa Christine Beermann, Kristina Alexanderson, Anna Martling, Lingjing Chen

**Affiliations:** 1 Division of Insurance Medicine, Department of Clinical Neuroscience, Karolinska Institutet, SE-171 77 Stockholm, Sweden; 2 Department of Molecular Medicine and Surgery, Karolinska Institutet, SE-171 77 Stockholm, Sweden

**Keywords:** Sick leave, Disability pension, Work loss, Cancer survivorship, Colorectal cancer

## Abstract

**Purpose:**

To longitudinally investigate overall and diagnosis-specific sickness absence (SA) and disability pension (DP) in colorectal cancer (CRC) survivors and references and to identify potential risk factors.

**Methods:**

This longitudinal register-based cohort study included all patients living in Sweden, diagnosed with a first primary CRC in 2008–2011 when aged 18–62 (*n*=6679), and their matched references (*n*=26 716). Net days of SA (in SA spells >14 days) and DP were analyzed from 2 years before through 5 years after diagnosis, overall and by specific diagnoses. Among survivors, risk factors for future SADP were explored using logistic regression.

**Results:**

In survivors, SA peaked in year 1 postdiagnosis, with 62.5% having at least some SA, and then gradually decreased to 20.1% in year 5. In the 2 years after diagnosis, CRC was the most common SA diagnosis in survivors, while SA due to mental diagnoses remained similar to the references. Notable risk factors for postdiagnostic SA or DP were rectal cancer diagnosis, advanced cancer stage at diagnosis, lower educational level, born outside of Sweden, and pre-diagnostic SA, mental morbidity, and comorbidities.

**Conclusion:**

During 5 years after a CRC diagnosis, CRC survivors had higher levels of postdiagnostic SA and DP than the references, which was mostly due to CRC diagnoses. Although their SA lowered gradually, it did not return to pre-diagnostic levels.

**Implications for Cancer Survivors:**

Our results provide valuable information for patients with CRC diagnosis, especially that most have none or low levels of SA/DP after a few years.

**Supplementary Information:**

The online version contains supplementary material available at 10.1007/s11764-021-01017-7.

## Background

Colorectal cancer (CRC) is one of the most common cancer types, ranking third in cancer incidence and second in cancer mortality worldwide [1]. Recently, the incidence of CRC is increasing in high-income countries among people below 50 years [2, 3], while mortality declines [4, 5]. Hence, for growing numbers of working-age CRC survivors, future work-related outcomes are of rising importance.

Pursuing paid work after cancer diagnosis and treatment is a significant part of recovery, as it helps cancer survivors to regain control and normalcy in their lives [6–10]. However, CRC, especially colon cancer survivors, is reported to have reduced capacity for paid work after diagnosis [11–14]. Risk factors for future sickness absence (SA) and disability pension (DP) include comorbidities, previous SA, advanced cancer stage, chemotherapy, radiotherapy, extensive surgery, and postoperative complications, with inconsistent findings for the influence of educational level [11, 12, 15].

Most previous studies on CRC survivors’ work capacity have focused on the binary outcome return-to-work (measured as yes/no) [13, 14]. Furthermore, these studies only have a short follow-up, not accounting for long-term effects that CRC diagnosis and treatment may have on survivors’ work capacity. The few previous studies that used SA and DP as an outcome used data from patients diagnosed 1992–2005 and aged 45–54 at diagnosis [11], or measured SA as a binary outcome (yes/no) and not using a reference group [12], or only including rectal cancer survivors [15, 16].

Moreover, CRC survivors’ specific SA and DP diagnoses have not yet been investigated. Such knowledge, in comparison to the general population, will enable clinicians to better understand occurrences of behind future long-term SA and even DP in survivors. Hence, there is a need of longitudinal studies using recent data and a reference group to provide comprehensive information on CRC survivors’ long-term SA and DP.

Therefore, we aimed to (1) longitudinally investigate overall and diagnosis-specific SA and DP in CRC survivors and in their matched references and (2) identify possible risk factors for overall SA and DP.

## Methods

A longitudinal cohort study of first primary CRC patients and matched references was conducted.

Data from the following six nationwide Swedish registers were linked on an individual level through the personal identity numbers assigned to all residents in Sweden [17].

*-National Board of Health and Welfare*: Swedish Cancer Register [18] for the identification of cancer diagnosis, diagnosis date, and cancer stage; National Patient Register [19] for inpatient and specialized outpatient visits; Cause of Death Register [20] for death date; and Prescribed Drug Register [21] for dispensed prescribed psychiatric medication
*-Statistics Sweden*: Longitudinal Integrated Databases for Health Insurance and Labour Market Studies [22] (LISA) for socio-demographic information including sex, birth year, educational level, birth country, and emigration
*-Swedish Social Insurance Agency*: Microdata for Analyses of Social Insurance [23] (MiDAS) for all DP and all SA spells >14 days regarding start/end date, grade (full- or part-time), and main diagnosis (according to International Classification of Diseases, the tenth revision, ICD-10) [24]

### Inclusion and exclusion criteria

We included all people diagnosed with a first primary CRC in Sweden in 2008–2011 (not diagnosed at autopsy) when aged 18–62 (*N*=6679). We used ICD-10 codes C18 and C19–20 to identify colon and rectal cancer, respectively. From LISA, 26,716 population references were matched to the patients regarding sex, age, birth country, and educational level (four references per patient). References were selected randomly and were alive and without previous record of CRC before the diagnosis date of the index person and registered as living in Sweden the year before the diagnosis year of the index person and alive at inclusion. Survivors and references were followed from diagnosis date through 5 years later (e.g., Y_+1_ = diagnosis date + 365 days). Individuals were censored from the year following their death, emigration, or turning 65 (old-age pension age), whichever came first.

The reason for this is that they were no longer at risk for the outcome (SA/DP) afterwards. A detailed flow chart was presented (Online Resource 1).

### The Swedish public sickness absence and disability pension benefit scheme

In Sweden, SA can be granted to residents ≥16 years with an income from work or unemployment benefit, if their work capacity is reduced due to disease or injury. A physician medical certificate is needed from day 8. For employees, the first 2 weeks of SA benefits are paid by employers, afterwards by the Social Insurance Agency. For the unemployed, the Social Insurance Agency pays from day 2. Therefore, we only used information on SA spells >14 days registered by the Social Insurance Agency. In most of the studied years, there was no maximum duration of a SA spell. In some of the years, there was a limit of 914 days (2.5 years), followed by a waiting period of 3 months before SA benefits could be claimed again. Temporary or permanent DP can be granted to people aged 19–64 years with long-term or permanent work incapacity due to disease or injury [25].

SA and DP benefits cover 80% and 64% of lost income, respectively, up to a certain level. SA and DP can be granted for full- or part-time (100%, 75%, 50%, or 25%) of ordinary work hours [25]; thus, people can have both partial SA and DP at the same time.

### Outcome

The main outcomes were SA and DP following the CRC diagnosis date. The SA and DP net days/year during the follow-up were calculated for survivors and their matched references. Net days were calculated by multiplying the degree of compensation with the number of compensated days: e.g., two gross days of 50% SA or DP equaled one net day (net days are from here on called as only days).

SA days were further categorized into 0, >0–30, >30–90, >90–180, and >180 days/year. DP was dichotomized in 0 and >0 days/year. As the majority of SA and DP days in Sweden are due to mental and musculoskeletal diagnoses [25, 26], we categorized medical diagnoses for SA and DP days as CRC, mental, musculoskeletal, or other diagnoses.

### Characteristics

Sociodemographic variables included sex, age at diagnosis, birth country, and educational level (detailed in Table 1).

**Table 1:** Sociodemographic and clinical characteristics of colorectal cancer survivors diagnosed in 2008–2011 when aged 18–62 years and of their matched references

Characteristics	Colorectal cancer survivors no. (%)	References no. (%)
All	6679 (100)	26,716 (100)
Sex		
Men	3598 (53.9)	14,392 (53.9)
Women	3081 (46.1)	12,324 (46.1)
Age, years		
18–50	1783 (26.7)	7132 (26.7)
51–55	1319 (19.8)	5276 (19.8)
56–60	2271 (34.0)	9084 (34.0)
61–62	1306 (19.6)	5224 (19.6)
Country of birth		
Sweden	5624 (84.2)	22,496 (84.2)
other	1055 (15.8)	4220 (15.8)
Educational level (years)		
Elementary school (<10)	1467 (22.0)	5868 (22.0)
High school (10-12)	3120 (46.7)	12,480 (46.7)
University/college (>12)	2092 (31.3)	8368 (31.3)
Cancer type		
Colon cancer	4044 (60.6)	-
Rectal cancer	2635 (39.5)	-
Cancer stage		
Stage 0 + I	2109 (31.6)	-
Stage II	1270 (19.0)	-
Stage III	1464 (21.9)	-
Stage IV	1203 (18.0)	-
Missing	633 (9.5)	-
Charlson Comorbidity Index^a^ in the 3 years prior to diagnosis date (Y_-3_ – Y_-1_)		
0+1	5505 (82.4)	25,588 (95.8)
≥2	1174 (17.6)	1128 (4.2)
Mental morbidity in the 3 years prior to diagnosis date (Y_-3_ – Y_-1_)		
None	5424 (81.2)	22,317 (83.5)
Any	1255 (18.8)	4399 (16.5)

^a^Charlson Comorbidity Index was calculated based on all diagnoses from specialized healthcare within the 3 years before diagnosis date

Stage was classified into stage 0, I, II, III, IV, and missing, based on the information of T (tumor size), N (lymph nodes), and M (metastases) [27] from the Swedish Cancer Register. If T and/or N and M were missing or classified as X (assessment not possible), stage was classified as missing. If more than one entry of the same type of cancer diagnosis was found in the register within 30 days, the most advanced stage was used.

The Charlson Comorbidity Index (CCI) [28] at diagnosis was calculated for survivors and their references based on the National Patient Register regarding inpatient and specialized outpatient visits during all the 3 years before diagnosis date. Based on the same register, pre-diagnostic mental morbidity was defined as having any healthcare during the 3-year period before diagnosis date with ICD-10 codes of “F00-F99” or “Z73” or having any prescribed psychiatric medication for depression, anxiety, tension, or psychotics, according to the prescribed drug register.

### Statistical analysis

The numbers and percentages of people with different levels of annual SA and DP days were computed from the second year before (Y_-2_) through the fifth year after (Y_+5_) diagnosis date for both cohorts. The mean SA and DP days/year overall and by specific SA/DP diagnoses were calculated.

For CRC survivors, univariable and multivariable logistic regression were used to examine the associations between covariates and future SA and DP, respectively. In all logistic regression models, pre-diagnostic SA was assessed by using SA in Y_-2_. Therefore, survivors not living in Sweden in Y_-2_ were excluded in those analyses.

In the analyses of SA risk, the odds ratios (OR) with 95% confidence intervals (CI) of having >30 days of SA in Y_+3_ and Y_+5_, respectively, were estimated. For specific years, survivors were excluded from the analyses if they died or emigrated before or had full-time DP during all of the respective years. For Y_+5_, survivors aged 61–62 at diagnosis were also excluded because of possible transition into old-age pension. In the analyses of DP risk during the follow-up period, survivors on any DP in Y_-1_ and those who died or emigrated during follow-up without having been granted DP were excluded.

All statistical analyses were performed with STATA version 14. For all tests, the level of significance was set at *p*<0.05.

The project was approved by the Regional Ethical Review Board in Stockholm.

## Results

In both CRC survivors (*n*=6679) and references (*n*=26,716), the majority were men (53.94%) and born in Sweden (84.2%), and 34.0% were diagnosed when aged 56–60 (Table 1). Survivors and their references differed regarding the distribution of pre-diagnostic CCI (17.6% of survivors vs. 4.2% of references with CCI score ≥2). Among survivors, 60.6% had a colon cancer diagnosis, and the most common cancer stage was stage 0+I (31.6%). The corresponding baseline characteristics stratified by colon and rectal cancer survivors are presented in Online Resource 2.

### Distribution of annual SA and DP

SA and DP days/year in survivors and in their matched references are presented by the different categories (Table 2). Among the references, on average 10.6% had some SA during the observation period. The proportion of CRC survivors with SA was 12.8% during Y_-2_ and increased to 30.2% in Y_-1_. In Y_+1_, 62.5% of survivors had some SA, half of them for >180 days. This proportion decreased to 37.5% in Y_+2_ and further to 20.1% in Y_+5_ but did not reach the pre-diagnostic levels. The proportion of survivors with any SA in Y_+5_ was 8.8% higher than in the reference group. Among survivors, 18.6% had some DP in Y_-2_ compared to 16.0% among the references. These proportions decreased to 17.3% and 13.4%, respectively, in Y_+5_, with a difference of 3.8%. (If excluding references for which the patient had died or emigrated during follow-up, figures were nearly exactly the same.)

**Table 2:** Annual distribution of sickness absence and disability pension, respectively, among colorectal cancer survivors and their matched references

		Colorectal cancer survivors							Matched references						
		Before diagnosis date		After diagnosis date					Before diagnosis date		After diagnosis date				
	No. of days	Year -2, no. (%)	Year -1, no. (%)	Year +1, no. (%)	Year +2, no. (%)	Year +3, no. (%)	Year +4, no. (%)	Year +5, no. (%)	Year -2, no. (%)	Year -1, no. (%)	Year +1, no. (%)	Year +2, no. (%)	Year +3, no. (%)	Year +4, no. (%)	Year +5, no. (%)
All included		6661 (100)	6 679 (100)	6679 (100)	6025 (100)	5553 (100)	4713 (100)	4005 (100)	26,613 (100)	26,716 (100)	26,716 (100)	26,493 (100)	26,331 (100)	23,574 (100)	20,953 (100)
Sickness absence	0	5806 (87.2)	4663 (69.8)	2506 (37.5)	3764 (62.5)	4134 (74.5)	3666 (77.8)	3200 (79.9)	23,606 (88.7)	23,869 (89.3)	23,983 (89.8)	23,801 (89.8)	23,720 (90.1)	21,096 (89.5)	18,579 (88.7)
	>0–30	293 (4.4)	1280 (19.2)	295 (4.4)	409 (6.8)	258 (4.7)	173 (3.7)	164 (4.1)	995 (3.7)	981 (3.7)	937 (3.5)	904 (3.4)	858 (3.3)	736 (3.1)	685 (3.3)
	>30–90	268 (4.0)	420 (6.3)	1004 (15.0)	568 (9.4)	362 (6.5)	293 (6.2)	217 (5.4)	950 (3.6)	953 (3.6)	910 (3.4)	898 (3.4)	849 (3.2)	795 (3.4)	757 (3.6)
	>90–180	142 (2.1)	165 (2.5)	720 (10.8)	472 (7.8)	293 (5.3)	222 (4.7)	165 (4.1)	494 (1.9)	455 (1.7)	449 (1.7)	457 (1.7)	422 (1.6)	451 (1.9)	417 (2.0)
	>180	152 (2.3)	151 (2.3)	2154 (32.3)	812 (13.5)	506 (9.1)	359 (7.6)	259 (6.5)	568 (2.1)	458 (1.7)	437 (1.6)	433 (1.6)	482 (1.8)	496 (2.1)	515 (2.5)
Disability pension	0	5421 (81.4)	5413 (81.1)	5393 (80.8)	4901 (81.3)	4574 (82.4)	3906 (82.9)	3313 (82.7)	22,351 (84.0)	22,365 (83.7)	22,332 (83.6)	22,144 (83.6)	22,406 (85.1)	20,268 (86.0)	18,136 (86.6)
	>0	1240 (18.6)	1266 (19.0)	1286 (19.3)	1124 (18.7)	979 (17.6)	807 (17.1)	692 (17.3)	4262 (16.0)	4351 (16.3)	4384 (16.4)	4349 (16.4)	3925 (14.9)	3306 (14.0)	2817, (13.4)
Not included^a^		18	-	-	654	1126	1966	2674	103	-	-	223	385	3142	5763

Number and percentages of colorectal cancer survivors and references having different numbers of sickness absence or disability pension net days per year during the 2 years before through the 5 years after the date of colorectal cancer diagnosis (for references, diagnosis date of matched patient was used)

^a^Individuals were included up to and including the year they turned 65 years of age, died, or emigrated

### Diagnosis-specific SA and DP

**Fig. 1:**
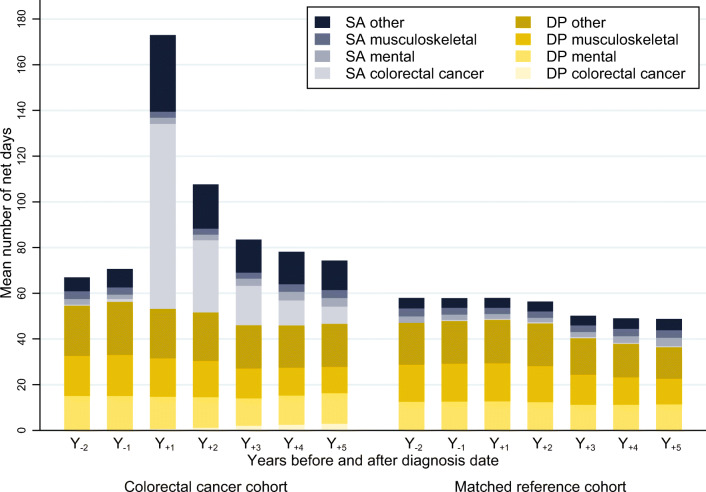
Mean number of sickness absence (SA) and disability pension (DP) net days per year and by diagnosis for colorectal cancer survivors and for their matched references. Mean number of sickness absence (SA) and disability pension (DP) net days per yearly interval from the second year before (Y_-2_) until the fifth year after (Y_+5_) the date of colorectal cancer diagnosis (for references, diagnosis date of matched patient was used). The survivors and references were not included anymore after turning 65 years of age, death, or emigration. In Y_-2_, people not yet living in Sweden at that time were not included. In the colorectal cancer cohort, the total number of people included in the analyses from Y_-2_ to Y_+5_ were 6661, 6679, 6679, 6025, 5553, 4713, and 4005, respectively. For the matched reference cohort, the corresponding numbers were 26613, 26716, 26716, 26493, 26331, 23574, and 20953

Before diagnosis, overall mean combined SA and DP days/year were slightly higher among CRC survivors compared to among references (67.0 days/year in survivors vs. 57.9 days/year in references in Y_-2_) (Fig. 1). After diagnosis, mean SA days in survivors peaked in Y_+1_ (119.8 days/year) and then drastically dropped to 56.0 days in Y_+2_ and further to 27.7 days in Y_+5_. In contrast, the mean SA days in references remained stable during the postdiagnostic period (9.1–11.9 days/year).

Opposed to mean SA days, mean DP days after diagnosis were more comparable in both groups and decreased slightly with time during follow-up (from 53.2 to 46.6 days/year in survivors vs. from 48.8 to 36.8 days/year in references). In Y_+5_, the survivors still had higher DP rates, and the difference between survivors and references in overall mean SA was 15 days and in mean DP: 9 days.

Regarding the specific SA and DP diagnoses, among CRC survivors, 68% of all mean SA days in Y_+1_ were due to CRC (81 mean days) and 27% in Y_+5_(7.5 mean days). Meanwhile, the proportion of mean DP days due to CRC increased from 1.1% in Y_+1_ (0.6 mean days) to 6.1% in Y_+5_(2.9 mean days). Comparing survivors and references, mean SA and DP days/year due to mental or musculoskeletal diagnoses were similar in both groups during the postdiagnostic follow-up. Mean SA days due to mental diagnoses amounted to 2.5 in survivors and 2.8 in references in Y_-2_ and increased in both cohorts during follow-up to 3.9 and 3.6 mean SA days in Y_+5_, respectively. Mean DP days with mental diagnoses ranged between 12.0 and 15.0 days in survivors and 11.2 and 12.7 days in references. Regarding musculoskeletal diagnoses, survivors had 3.4 and references 3.5 mean SA days in Y_-2_ which stayed constant during follow-up, while mean DP days ranged between 11.5–18.0 among survivors and 11.2–16.7 among references. (The figure did not change if excluding references for which the patient had died or emigrated during follow-up.)

However, the number of mean SA and DP days for the diagnostic group “other” was slightly higher in survivors than in their references. While mean SA days/year varied between 4.2 and 4.9 days in references, survivors had 33.5 mean SA days due to other diagnoses in Y_+1_, which decreased to 12.9 in Y_+5_. Concerning DP, mean days with other diagnoses in Y_-2_ were 22.3 in survivors and 18.3 in references and decreased to 18.8 and 14.2 mean days in Y_+5_, respectively. Diagnosis-specific mean SA and DP days/year are also presented by colon and rectal cancer survivors (Online Resource 2). The percentages were similar, although somewhat higher among rectal cancer survivors in the postdiagnostic years.

### Risk for future sickness absence among CRC survivors

In Y_+3_ and Y_+5_, 23.0% and 17.6% of CRC survivors, not on full-time DP, had >30 SA days, respectively (Table 3). After adjustments, in Y_+3_, the strongest association with future SA >30 days could be seen for cancer stage II–IV, with stage IV having an OR of 10.3 (95% CI 8.1–13.2) compared to stage 0+1. Those with SA >30 days in Y_-2_ also had higher ORs. Having pre-diagnostic SA >180 days compared to 0 such days rendered an OR of 2.8 (95% CI 1.8–4.3). Other factors with a substantially higher likelihood for SA >30 days in Y_+3_ were rectal cancer diagnosis and pre-diagnostic CCI ≥2. A lower likelihood on the other hand was found among survivors aged 61–62 at diagnosis compared to 56–60.

**Table 3:** Crude and adjusted odds ratios (OR) with 95% confidence intervals (CI) for having sickness absence >30 net days during year 3 (Y_+3_) and 5 (Y_+5_) postdiagnosis, respectively, and for being granted disability pension during the 5-year postdiagnosis period, among colorectal cancer survivors

	Sickness absence year 3 (Y_+3_) (*n* = 5044)			Sickness absence year 5 (Y_+5_) (*n* = 3641)			Disability pension during 5-year follow-up		
Variable and categories	On sickness absence/all included survivors at risk (%)	Crude OR (95% CI)	Adjusted OR (95% CI)	On sickness absence/all included survivors at risk (%)	Crude OR (95% CI)	Adjusted OR (95% CI)	Granted disability pension/all included survivors at risk (%)	Crude OR (95% CI)	Adjusted OR (95% CI)
Total	1161/5044 (23.0)			640/3641 (17.6)			229/3981 (5.8)		
Sex									
Men	607/2704 (22.5)	1	1	304/1902 (16.0)	1	1	125/2164 (5.8)	1	1
Women	554/2340 (23.7)	1.07 (0.94–1.22)	1.04 (0.90–1.20)	336/1739 (19.3)	**1.26 (1.06–1.50)**	**1.20 (1.00–1.43)**	104/1817 (5.7)	0.99 (0.76–1.30)	0.87 (0.65–1.17)
Age (years)									
18–50	372/1390 (26.8)	1.11 (0.94–1.30)	1.15 (0.96–1.37)	229/1216 (18.8)	**1.37 (1.12–1.68)**	**1.44 (1.17–1.77)**	72/1148 (6.3)	0.91 (0.66–1.26)	1.02 (0.72–1.45)
51–55	269/1001 (26.9)	1.11 (0.93–1.33)	1.17 (0.97–1.42)	188/884 (21.3)	**1.60 (1.29–1.98)**	**1.69 (1.35–2.10)**	55/807 (6.8)	1.00 (0.70–1.42)	1.12 (0.77–1.63)
56–60	399/1607 (24.8)	1	1	223/1541 (14.5)	1	1	87/1274 (6.8)	1	1
61–62	121/1046 (11.6)	**0.40 (0.32–0.49)**	**0.36 (0.29–0.46)**	-	-	-	15/752 (2.0)	**0.28 (0.16–0.48)**	**0.25 (0.14–0.44)**
Country of birth									
Sweden	975/4283 (22.8)	1	1	541/3078 (17.6)	1	1	173/3387 (5.1)	1	1
Other	186/761 (24.4)	1.10 (0.92–1.31)	1.12 (0.92–1.36)	99/563 (17.6)	1.00 (0.79–1.27)	1.06 (0.83–1.35)	56/594 (9.4)	**1.93 (1.41–2.65)**	**1.85 (1.31–2.61)**
Educational level (years)									
Elementary school (<10)	224/998 (22.4)	1.05 (0.88–1.28)	1.14 (0.93–1.40)	109/640 (17.0)	1.08 (0.84–1.39)	1.11 (0.85–1.44)	52/729 (7.1)	**1.69 (1.16–2.47)**	**1.58 (1.05–2.39)**
High school (10–12)	566/2320 (24.4)	**1.18 (1.02–1.37)**	**1.21 (1.03–1.42)**	323/1697 (19.0)	**1.24 (1.02–1.50)**	**1.24 (1.02–1.52)**	115/1824 (6.3)	**1.48 (1.08–2.04)**	**1.42 (1.01–1.98)**
University/college (>12)	371/1726 (21.5)	1	1	208/1304 (16.0)	1	1	62/1428 (4.3)	1	1
Cancer type									
Colon cancer	627/2984 (21.0)	1	1	369/2180 (16.9)	1	1	132/2355 (5.6)	1	1
Rectal cancer	534/2060 (25.9)	**1.32 (1.15–1.50)**	**1.48 (1.28–1.70)**	271/1461 (18.6)	1.12 (0.94–1.33)	1.16 (0.97–1.39)	97/1626 (6.0)	1.07 (0.82–1.40)	1.20 (0.90–1.60)
Cancer stage									
Stage 0 + I	249/1835 (13.6)	1	1	197/1416 (13.9)	1	1	64/1568 (4.1)	1	1
Stage II	227/1081 (21.0)	**1.69 (1.39–2.06)**	**1.80 (1.47–2.21)**	130/831 (15.6)	1.15 (0.90–1.46)	1.22 (0.95–1.55)	47/909 (5.2)	1.28 (0.87–1.88)	1.43 (0.94–2.15)
Stage III	323/1203 (26.9)	**2.34 (1.94–2.81)**	**2.50 (2.06–3.02)**	175/849 (20.6)	**1.61 (1.28–2.01)**	**1.71 (1.36–2.15)**	57/920 (6.2)	**1.55 (1.08–2.24)**	**1.88 (1.28–2.78)**
Stage IV	269/453 (59.4)	**9.31 (7.40–11.72)**	**10.34 (8.10–13.18)**	75/164 (45.7)	**5.21 (3.70–7.34)**	**5.54 (3.90–7.87)**	44/188 (23.4)	**7.18 (4.72–10.93)**	**9.57 (6.00–15.26)**
Missing	93/472 (19.7)	**1.56 (1.20–2.03)**	**1.53 (1.17–2.00)**	63/381 (16.5)	1.23 (0.90–1.67)	1.23 (0.90–1.68)	17/396 (4.3)	1.05 (0.61–1.82)	1.12 (0.63–1.98)
Charlson Comorbidity Index in the 3 years prior to diagnosis date (Y_-3_ – Y_-1_)									
0+1	941/4394 (21.4)	1	1	559/3283 (17.0)	1	1	174/3572 (4.9)	1	1
≥2	220/650 (33.9)	**1.88 (1.57–2.24)**	**1.52 (1.24–1.85)**	81/358 (22.6)	**1.43 (1.10–1.86)**	1.25 (0.95–1.65)	55/409 (13.5)	**3.03 (2.20–4.19)**	**2.65 (1.86–3.78)**
Mental morbidity in the 3 years prior to diagnosis date (Y_-3_ – Y_-1_)									
None	949/4273 (22.1)	1	1	508/3090 (16.4)	1	1	159/3465 (4.6)	1	1
Any	212/771 (27.5)	**1.33 (1.12–1.58)**	**1.34 (1.10–1.63)**	132/551 (24.0)	**1.60 (1.29–2.00)**	**1.55 (1.23–1.97)**	70/516 (13.6)	**3.26 (2.42–4.40)**	**2.46 (1.74–3.49)**
No. of sickness absence days in the second year before diagnosis date (Y_-2_)									
0	948/4386 (21.6)	1	1	523/3.167 (16.5)	1	1	161/3.488 (4.6)	1	1
>0–30	63/235 (26.8)	1.33 (0.99–1.79)	1.26 (0.91–1.74)	41/183 (22.4)	**1.46 (1.02–2.09)**	1.41 (0.97–2.05)	9/179 (5.0)	1.09 (0.55–2.18)	0.89 (0.43–1.83)
>30–90	68/218 (31.2)	**1.64 (1.22–2.21)**	**1.84 (1.34–2.54)**	42/155 (27.1)	**1.88 (1.30–2.71)**	**1.76 (1.20–2.58)**	17/166 (10.2)	**2.36 (1.39–3.99)**	**1.83 (1.04–3.25)**
>90–180	41/103 (39.8)	**2.40 (1.61–3.58)**	**2.63 (1.69–4.08)**	13/62 (21.0)	1.34 (0.72–2.41)	1.16 (0.61–2.21)	14/75 (18.7)	**4.74 (2.60–8.66)**	**4.25 (2.22–8.14)**
>180	41/102 (40.2)	**2.44 (1.63–3.65)**	**2.75 (1.78–4.27)**	21/74 (28.4)	**2.00 (1.20–3.35)**	**1.81 (1.05–3.10)**	28/73 (38.4)	**12.86 (7.82–21.15)**	**9.55 (5.43–16.79)**

Bold text indicates a statistically significant association with both sided *p*<0.05. In adjusted models, all covariates (sex, age, country of birth, educational level, cancer type, cancer stage, Charlson Comorbidity Index, mental morbidity, pre-diagnostic sickness absence) were included

*CI* confidence interval, *OR* odds ratio, *DP* disability pension

In Y_+5_, similar patterns of risk indicators for having SA >30 days were observed. Diagnosed with stage IV rendered an adjusted OR of 5.5 (95% CI 3.9–7.9) and pre-diagnostic SA >180 days an OR of 1.8 (95% CI 1.1–3.1). Additionally, pre-diagnostic mental morbidity or being diagnosed at a younger age (18–55 vs. 56–60 years) implied a higher risk of SA >30 days in Y_+5_.

### Risk for future disability pension among CRC survivors

Among those at risk for DP (i.e., excluding those already on DP before diagnosis date and those who during the follow-up died or emigrated before being granted DP), 5.8% of the survivors were granted DP during the 5-year follow-up (Table 3). After adjustments, the OR for DP was significantly higher among survivors with stage III (OR 1.9; 95% CI 1.3–2.8) and stage IV cancer (OR 9.6; 95% CI 6.0–15.3) compared to those with stage 0+I. Those with any pre-diagnostic SA compared to none in Y_-2_ also had higher risk of being granted DP postdiagnosis, especially among those with pre-diagnostic SA >180 days (OR 9.6; 95% CI 5.4–16.8). Other risk factors for DP were pre-diagnostic mental morbidity, pre-diagnostic CCI ≥2, lower educational level, and being born outside of Sweden, while those diagnosed when aged 61–61 had lower risk compared to those diagnosed when 56–60.

## Discussion

In this large Swedish longitudinal cohort study of 6679 CRC survivors, the proportion of survivors on SA and DP after their diagnosis was significantly higher than among their matched references from the general population. Nevertheless, following the expected peak during the first year postdiagnosis, a clear decline was seen over the 5-year follow-up period. Most survivors (65%) did not have any SA or DP benefits in the fifth year postdiagnosis. However, SA in survivors did not decrease to pre-diagnostic levels and remained higher than in references. The risk for having SA and DP during follow-up was highest among survivors with advanced cancer stage and prior SA.

Our main finding of higher levels of SA and DP in CRC survivors than in references, with a peak of SA in Y_+1_, are in line with results from two previous studies [11, 15], implying that treatment-related factors may have the strongest impact on SA in the first year postdiagnosis.

Uniquely, we also explored the SA and DP diagnoses and showed that CRC was the most common diagnosis for SA days in survivors postdiagnostically until Y_+3_, accounting for 68% of all mean SA days in Y_+1_ and 27% in Y_+5_. Meanwhile, the proportion of mean DP days due to CRC gradually increased to 6% in Y_+5_ from 1% in Y_+1_.

No major differences were observed between the survivors and references regarding SA and DP days due to mental or musculoskeletal diagnoses in any of the studied years. Concerning mental morbidity following CRC diagnoses, literature presents mixed results: reduced mental health after diagnosis compared to in the general population was found in some studies, while no difference was reported in others [29]. Here we had no information on mental disorders following diagnoses; however, SA/DP due to mental diagnoses did not increase, possibly indicating that mental disorders due to the CRC diagnoses were not severe enough to lead to severe work incapacity. More knowledge is needed about this.

The SA/DP diagnostic category “other diagnoses” caused most of the remaining difference between survivors and references in mean SA and DP days/year. Part of this difference could be explained by the diagnosis of secondary cancers, since 18% of included survivors already had stage IV at diagnosis, and in general, one-third of CRC survivors experience cancer relapse (most within 3 years of diagnosis) [30].

In line with three other studies [11, 12, 15], we found that those with advanced cancer stage, especially stage IV, had a higher risk of future SA and DP than those with stage 0+I, probably due to more severe disease and more aggressive and/or longer treatment. This potential association is supported by studies showing lower work capacity or return-to-work in CRC patients treated with chemotherapy, radiotherapy, and extensive surgery [12–15, 31].

As also found in two previous studies [11, 12], rectal cancer survivors had higher odds for SA in Y_+3_ compared to colon cancer survivors. Reasons could be more aggressive treatment strategies like neoadjuvant chemo- and radiotherapy, abdominoperineal resection with the necessity of a stoma [14, 15], and higher rates of postoperative complications in these patients [32].

We found that high comorbidity score (CCI ≥2), prior mental morbidity, and having more pre-diagnostic SA days all rendered a higher risk of SA and DP postdiagnosis. These findings imply that morbidity prior to diagnosis plays an important role in future SA and DP, something also supported by previous studies [11, 12, 15]. Furthermore, mental morbidity is associated with higher somatic morbidity [33–35] and may therefore imply higher risk of future SA and DP not only through mental but also somatic disorders.

Our results indicate that those with no university/college education have higher risk of SA and DP. Possible reasons for this could be an association between lower educational level and later physically demanding jobs in less flexible workplaces [36, 37]. Previous findings about associations with educational level have been inconclusive [31]. Further research could investigate how occupation-related factors are associated with future SA and DP. The risk for SA and DP was lower in higher ages, as people transitioned into retirement age and some might also have taken early old-age pension. Notably, the CRC cohort had higher levels of SA/DP days already 2 years before diagnosis and a higher comorbidity score during the 3 years before diagnosis.

## Strengths and limitations

Strengths of this study include the population-based, longitudinal design using nationwide registers of high quality and completeness [18, 19, 25], avoiding selection bias and loss to follow-up, that all, not only employed, were included and that data on SA and DP diagnoses could be included. The large study population allowed for sub-group analyses. Another strength is that all were followed from actual diagnosis date, instead of calendar year. In the analyses, we excluded people not anymore at risk for the outcome, i.e., after they died, emigrated, or turned 65. It is a strength that we had information on this; if keeping people not at risk, the SA/DP rates would falsely have seemed to be lower. However, if some of those who died due to, e.g., late diagnosis, might have survived longer if diagnosed earlier, it might have been so that SA rates might have been higher. The results are representative of the population in Sweden and can be generalized to other countries with similar welfare systems.

Several limitations were noted also. SA spells ≤14 days were not included, possibly leading to an underestimation of annual SA days. However, by using a comparison group, we limited the influence of this. Furthermore, by including only SA spells >14 days, our outcome reflects long-term, more severe SA spells. Another limitation was that treatment data was not available. Still, the information about cancer stage may partially compensate for this. Information about cancer relapse was also not included, possibly causing an overestimation of other factors’ influence on the risk for SA and DP. Information about cancer stage was missing in 9.5% of cases, but comparable results in the analyses were found when excluding all missing data.

## Conclusion

This register-based longitudinal cohort study showed that CRC survivors were more likely to have SA and DP than their matched references, even before diagnosis. Although SA and DP in survivors decreased over time, number of SA and DP days was still higher after 5 years and had not returned to pre-diagnostic levels. The likelihood for long-term SA and DP was highest among survivors with more pre-diagnostic SA days and advanced cancer stage. These results emphasize the importance to support CRC survivors in improving work-related capacity, especially among those at high risk for long-term SA and DP, and indicate the relevance of early cancer diagnosis to prevent long-term work incapacity.

## Supplementary information

ESM 1 (PDF 77 kb)

ESM 2 (PDF 88 kb)

ESM 3 (PDF 93 kb)
