# Supplementary material for: Overall and diagnosis-specific sickness absence and disability pension in colorectal cancer survivors and references in Sweden
Source: J Cancer Surviv. 2021 Mar 16;16(2):269–78. doi: 10.1007/s11764-021-01017-7 (PMC8964659; doi:10.1007/s11764-021-01017-7)
Supplement: Supplementary file 1 — (PDF 77 kb) [file 11764_2021_1017_MOESM1_ESM.pdf]

Journal of Cancer Survivorship

Overall and diagnosis-specific sickness absence and disability pension in colorectal cancer survivors and references: a Swedish register-based longitudinal cohort study

Author list: Luisa Christine Beermann MD[1]; Kristina Alexanderson PhD[1]; Anna Martling MD PhD[2]; Lingjing Chen MD MPH PhD[1]

Author's affiliation:
[1] Division of Insurance Medicine, Department of Clinical Neuroscience, Karolinska Institutet, SE-171 77 Stockholm, Sweden
[2] Department of Molecular Medicine and Surgery, Karolinska Institutet, SE-171 77 Stockholm, Sweden

Corresponding author:

Lingjing Chen
Division of Insurance Medicine
Department of Clinical Neuroscience
Karolinska Institutet
SE-171 77 Stockholm, Sweden
lingjing.chen@ki.se

Online Resource 1

Flowchart of inclusion and exclusion criteria during the observation period (two years before until five years after diagnosis date)

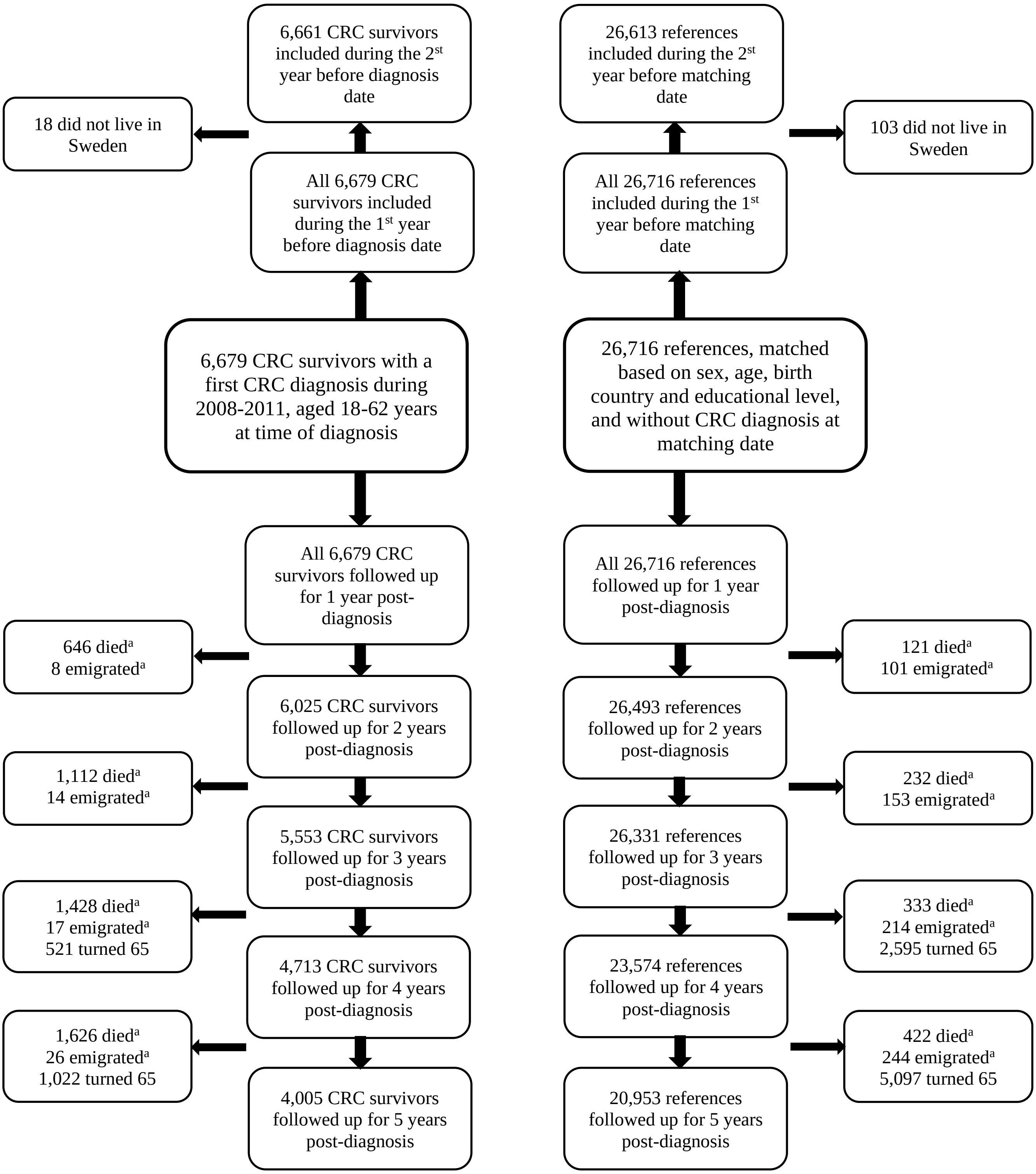

[a] before turning 65 years of age
