# Supplementary material for: Overall and diagnosis-specific sickness absence and disability pension in colorectal cancer survivors and references in Sweden
Source: J Cancer Surviv. 2021 Mar 16;16(2):269–78. doi: 10.1007/s11764-021-01017-7 (PMC8964659; doi:10.1007/s11764-021-01017-7)
Supplement: Supplementary file 2 — (PDF 88 kb) [file 11764_2021_1017_MOESM2_ESM.pdf]

Journal of Cancer Survivorship

Overall and diagnosis-specific sickness absence and disability pension in colorectal cancer survivors and references: a Swedish register-based longitudinal cohort study

Author list: Luisa Christine Beermann MD[1]; Kristina Alexanderson PhD[1]; Anna Martling MD PhD[2]; Lingjing Chen MD MPH PhD[1]

Author's affiliation:
[1] Division of Insurance Medicine, Department of Clinical Neuroscience, Karolinska Institutet, SE-171 77 Stockholm, Sweden
[2] Department of Molecular Medicine and Surgery, Karolinska Institutet, SE-171 77 Stockholm, Sweden

Corresponding author:

Lingjing Chen
Division of Insurance Medicine
Department of Clinical Neuroscience
Karolinska Institutet
SE-171 77 Stockholm, Sweden
lingjing.chen@ki.se

Online Resource 2. Sociodemographic and clinical characteristics of colon and rectal cancer survivors, respectively, diagnosed in 2008 - 2011 when aged 18-62 years

Characteristics	Colon cancer No. (%)	Rectal cancer No. (%)
**All**	4,044 (100)	2,635 (100)
**Sex**		
**Men**	2,134 (52.8)	1,464 (55.6)
**Women**	1,910 (47.2)	1,171 (44.4)
**Age (years)**		
**18-50**	1,103 (27.3)	680 (25.8)
**51-55**	765 (18.9)	554 (21.0)
**56-60**	1,384 (34.2)	887 (33.7)
**61-62**	792 (19.6)	514 (19.5)
**Country of birth**		
**Sweden**	3,399 (84.1)	2,225 (84.4)
**other**	645 (16.0)	410 (15.6)
**Educational level (years)**		
**Elementary school (<10)**	903 (22.3)	564 (21.4)
**High school (10-12)**	1,866 (46.1)	1,254 (47.6)
**University/College (>12)**	1,275 (31.5)	817 (31.0)
**Cancer type**		
**Colon**	-	-
**Rectal**	-	-
**Cancer stage**		
**Stage 0 + I**	1,126 (27.8)	983 (37.3)
**Stage II**	847 (20.9)	423 (16.1)
**Stage III**	919 (22.7)	545 (20.7)
**Stage IV**	777 (19.2)	426 (16.2)
**Missing**	375 (9.3)	258 (9.8)
**Charlson Comorbidity Index in the 3 years prior to diagnosis date (Y_{-3} – Y_{-1})**		
**0+1**	3,353 (82.9)	2,152 (81.7)
**≥2**	691 (17.1)	483 (18.3)
**Mental morbidity in the 3 years prior to diagnosis date (Y_{-3} – Y_{-1})**		
**No**	3,252 (80.4)	2,172 (82.4)
**Yes**	792 (19.6)	463 (17.6)
**No. of sickness absence days in the second year before diagnosis date (Y_{-2})**		
**0**	3,508 (86.8)	2,298 (87.2)
**>0-30**	172 (4.3)	121 (4.6)
**>30-90**	171 (4.2)	97 (3.7)
**>90-180**	83 (2.1)	59 (2.2)
**>180**	99 (2.5)	53 (2.0)
**Not living in Sweden**	11 (0.3)	7 (0.3)
**No. of disability pension days in the second year before diagnosis date (Y_{-2})**		
**0**	3,245 (80.2)	2,176 (82.6)
**>0**	788 (19.5)	452 (17.2)
**Not living in Sweden**	11 (0.3)	7 (0.3)
