# Supplementary material for: Overall and diagnosis-specific sickness absence and disability pension in colorectal cancer survivors and references in Sweden
Source: J Cancer Surviv. 2021 Mar 16;16(2):269–78. doi: 10.1007/s11764-021-01017-7 (PMC8964659; doi:10.1007/s11764-021-01017-7)
Supplement: Supplementary file 3 — (PDF 93 kb) [file 11764_2021_1017_MOESM3_ESM.pdf]

**Journal of Cancer Survivorship**

# Overall and diagnosis-specific sickness absence and disability pension in colorectal cancer survivors and references: a Swedish register-based longitudinal cohort study

**Author list:** Luisa Christine Beermann MD[1]; Kristina Alexanderson PhD[1]; Anna Martling MD PhD[2]; Lingjing Chen MD MPH PhD[1]

**Author's affiliation:**

[1] Division of Insurance Medicine, Department of Clinical Neuroscience, Karolinska Institutet, SE-171 77 Stockholm, Sweden
[2] Department of Molecular Medicine and Surgery, Karolinska Institutet, SE-171 77 Stockholm, Sweden

**Corresponding author:**

Lingjing Chen
Division of Insurance Medicine
Department of Clinical Neuroscience
Karolinska Institutet
SE-171 77 Stockholm, Sweden
lingjing.chen@ki.se

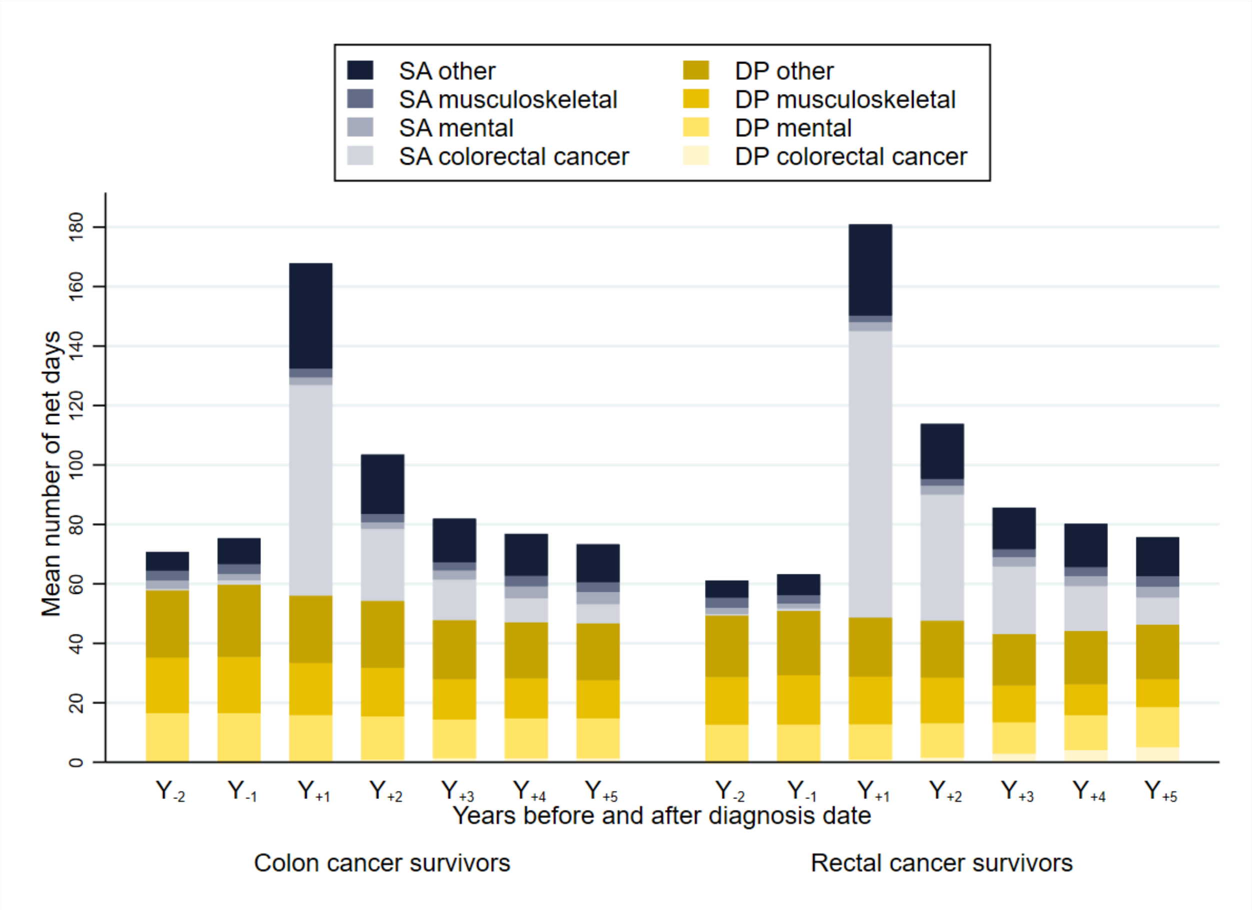


**Online Resource 3. Mean number of sickness absence (SA) and disability pension (DP) net days per year and by diagnosis for colon and for rectal cancer survivors, respectively.**

NOTE. Mean number of sickness absence (SA) and disability pension (DP) net days per yearly interval from the second year before ($Y_{-2}$) until the fifth year after ($Y_{+5}$) the date of colorectal cancer diagnosis. The survivors were not included anymore after turning 65 years of age, death, or emigration. In $Y_{-2}$, people not yet living in Sweden at that time were not included.

In the colon cancer cohort, the total number of people included in the analyses from $Y_{-2}$ to $Y_{+5}$ were 4033, 4044, 4044, 3597, 3302, 2806, and 2395, respectively. For the rectal cancer cohort, the corresponding numbers were 2628, 2635, 2635, 2428, 2251, 1907, and 1610.
